# The Chinese Druggist in New York*A paper read before the Alumni Association of the New York College of Pharmacy.

**Published:** 1882-11-20

**Authors:** Fred. Hohenthal


### THE CHINESE DRUGGIST IN NEW YORK.*

By FRED. HOHENTHAL.

The Chinese Drug-store here, the only one this side of San Francisco, was established by "Wwong Lung Iin," in 1878, and he is doing a flourishing business among his people now. The proprietor is a stout Chinaman, about 35 to 45 years of age, very intelligent, revered by his countrymen, but not so well versed in English as his assistant, who is about ten years younger. I experienced much trouble on my first visit in assuring him that I was not a customs officer ferreting out his opium trade, but on seeing my readiness to buy little trifles, he immediately installed me in his good graces, and invited me to dine with him.

The store is a small apartment, about 20x30 feet, with sleeping rooms, kitchen, parlor, etc., in the rear. There were shelves all around, covered with bottles and jars, and the top shelf mounted by many paper packages cabalistically marked, and containing roots and herbs in great variety. In a small case behind the counter were a few mineral drugs, and a good many little trifles not belonging strictly to Pharmacy, such as porcelain jars, pencils, skullcaps, beads, rings, etc. There were also some animal drugs of which I will speak later on.

Pendant from the ceiling were bunches of herbs and dried meat. On the floor, under the shelves, were articles of food, such as barrels of rice, flour, dried fish, etc. There were also about forty drawers behind the counter, for the most used drugs, those in the packages being only very seldom used. Large jars were filled with different pleasant tasting fruits, not used as medicines, and

*A paper read before the Alumni Association of the New York College of Pharmacy.

smaller glass jars with the various medicinal fruits, of which there was a large number, the chief uses of which seemed to be in the cure of consumption; a disease to which the Mongols seemed to be peculiarly liable, judging from the fact that about ten per cent of their medicines are for its cure.

They have no liquid medicines whatever; they simply sell the drugs, extolling their virtues, and the purchaser or patient prepares them with boiling water in the form of infusion or decoction and sometimes extract, unless he has not the conveniences therefor at home, when the apothecary will do it for him. As an accommodation for his customers, the doctor keeps a pot of hot tea on the counter; any one wishing to drink takes a cup from a basin of water near by, fills it, drinks and replaces the cup without saying a word. A peculiarly constructed tobacco pipe, on the principle of the Turkish narghileh, is also on the counter, but only for the use of intimate friends. The tobacco they use is very fine and of peculiar flavor. Their tobacco pipes, the common kind, are made of a rod of bamboo strung with the kernels of a peculiar scented nut, and furnished with a bowl of metal about half an inch in diameter and half an inch deep, and an ivory mouth-piece. The pipe is perfectly straight, and two feet long; they vary in price from $2.50 to $7.00 according to age; those which have been already smoked for some time bring more than new ones.

Their balances are on the plan of the steelyard, the small ones with a bar of ivory, from which is suspended a brass pan and a movable brass weight; one in my possession is twelve inches long and has over 125 marks for telling the weight, which ranges from two ounces downwards to two or three grains, very accurately. They have these balances of all sizes, and use them with great dexterity.

Their camel's hair pencils are about ten inches long, of bamboo, into one end of which the brush is inserted, and it is fitted with a cover, also of bamboo, the whole stem being curiously carved with their strange devices, and these cuts filled out with blue coloring.

Their writing materials are the pencil above described, an iron or porcelain plate six by eight inches, with a receptacle for water. They dip a piece of India ink in the water, rub it on the plate, and rub the pencil in this mixture, and write from above downwards.

They have some very pleasant fruits, one of these, the "Gua," is about the size of a walnut, and consists of a kernel as large as a hazelnut, surrounded by edible fleshy pulp, and the whole enveloped in a hard, brown, brittle shell.

Among the familiar drugs I found Spanish saffron, safflower, musk, litharge, metallic mercury, ginger, ginseng, oil of peppermint.

I found also Russian castor, and American castor, and what was claimed to be from the bear.

Also fine isinglass in one piece, just the size and shape of an ordinary flounder, and which he wanted to sell me for $2.00. He called it by a name that resembled "Guiteau."

I found also several narcotic herbs resembling belladonna, hyoscyamus, stramonium, also a root resembling glycyrrhiza, only much larger in diameter than that is usually found. It is called "Gum Cho," and is used for chewing on account of its sweet taste.

Among the most notable goods was a substance in small lumps of yellow color, and called "Nau Wau." It occurs in lumps, about the size of a walnut, in the stomachs of cows. The doctor said that it is found only in one cow in a hundred. It is used to apply to a sore foot in the form of paste, and is used only by the aristocracy.

There was a peculiar bark called "Os Chong," remarkable for the silky fibre it shows on breaking it in any direction; it is used in the form of decoction for weakness of the heart. The price is ten dollars a pound.

There were also dried lizards, which are to be boiled and eaten.

There were disinfecting fumigators, strips of bamboo, about one foot long, and as thick as a hairpin, which were covered on the upper half with a fragrant mass, which glowed for two hours when once lit, perfuming the rooms very pleasantly. They were called "Sau Hong."—*Drug. Cir.*

